# Prise en charge et résultats fonctionnels des cataractes traumatiques dans la région centrale du Togo

**DOI:** 10.11604/pamj.2016.25.107.7422

**Published:** 2016-10-24

**Authors:** Kassoula Batomaguela Nonon Saa, Nidain Maneh, Kokou Vonor, Mèba Banla, Ignace Sounouvou, Kossi Alaglo, Komi Patrice Balo

**Affiliations:** 1 Université de Lomé, Faculté des Sciences de la Santé, Ophtalmologie, Lomé, Togo; 2 Faculté des Sciences de la Santé de Cotonou, Ophtalmologie, UAC, Bénin; 3 Centre Hospitalier Régional (CHR) de Sokodé, Togo

**Keywords:** Cataracte traumatique, chirurgie, acuité visuelle, accessibilité, Togo, Traumatic cataract, surgery, visual acuity, accessibility, Togo

## Abstract

**Introduction:**

La cataracte traumatique est une affection grave, car pouvant être à l’origine de la baisse visuelle partielle ou totale. Le but de notre travail a été d'apprécier les résultats fonctionnels post opératoires des cataractes traumatiques opérées dans la région Centrale du Togo.

**Méthodes:**

Il s’agissait d’une étude rétrospective basée sur l’analyse des dossiers des patients ayant bénéficié d’une prise en charge chirurgicale pour cataracte traumatique en stratégie avancée et en poste fixe entre le 1er janvier 2009 et le 30 juin 2011.

**Résultats:**

Sur un total de 1086 cas de cataracte opérés au cours cette période, 131 (12,06%) étaient d’origine traumatique. La moyenne d’âge était de 30,01 ±16,61 ans avec des extrêmes de 5 et 70 ans 85% des patients ont bénéficié d’une implantation avec 66% de bons et moyens résultats après correction au premier jour postopératoire selon la classification de l’OMS. Après deux mois de suivi, 85% des patients revus présentaient une acuité visuelle supérieure à 3/10.

**Conclusion:**

Malgré les progrès de la micro chirurgie oculaire, celle de la cataracte traumatique demeure d’accès difficile à la fois pour le patient que pour le praticien surtout dans les pays en développement. Pour ces raisons la priorité doit être donnée à la prévention des traumatismes oculaires.

## Abstract (en)

**Introduction:**

Traumatic cataract is a serious condition because it can cause partial or total vision loss. Our study aims to assess the postoperative functional outcomes of patients undergoing surgery for traumatic cataract in the Central region of Togo.

**Methods:**

We conducted a retrospective study based on the analysis of medical records of patients who underwent surgical treatment for traumatic cataract in hospital based and outreach eye care services between 1 January 2009 and 30 June 2011.

**Results:**

Out of a total of 1086 cases with cataract undergoing surgery during this period, 131 (12.06%) had traumatic cataract. The average age was 30.01 ± 16.61 years, ranging betrween 5 and 70 years, 85% of patients underwent implantation with good or average success rates of 66% after correction in the first postoperative day (according to WHO classification). At two months follow up, 85% of patients had visual acuity above 3/10.

**Conclusion:**

Despite micro-ophthalmic surgery advances, access to traumatic cataract surgery remains difficult for both the patient and the practitioner, especially in developing countries. For these reasons priority must be given to ocular trauma prevention.

## Introduction

La cataracte traumatique est une affection grave, car pouvant être à l’origine de la baisse visuelle partielle ou totale [1–3]. Elle est préoccupante non seulement pour le patient mais aussi pour le praticien. Concernant les patients il faut noter que cette pathologie touche plus fréquemment les sujets jeunes en pleine activité avec son cortège de conséquences socio professionnelles. Les enfants ne sont pas épargnés et courent un grand risque d’amblyopie potentielle [4, 5]. Par rapport au praticien, l’œil traumatisé présente souvent des tableaux anatomo-cliniques variés qui rendent difficile sa prise en charge chirurgicale adéquate. La présente étude vise à apprécier les résultats fonctionnels post opératoires des cataractes traumatiques prises en charge dans les services d’ophtalmologie de la Région centrale du Togo.

## Méthodes

### Cadre d’étude

L’étude s’était déroulée dans les quatre préfectures de la Région Centrale du Togo (Blitta, Sotouboua, Tchamba et Tchaoudjo) et la préfecture de Bassar dans la région de la Kara. Dans cette zone, est mis en œuvre un projet de lutte contre la cécité par le Ministère de la Santé du Togo, soutenu par la Croix-Rouge Suisse dans le cadre d’un partenariat. La population de la zone du projet est estimée à environ 62% de la population totale.

### Type d’étude

Il s’agissait d’une analyse rétrospective de dossiers et comptes rendus opératoires de patients ayant bénéficié d’une prise en charge chirurgicale pour une cataracte traumatique entre le 1er janvier 2009 et le 30 juin 2011. Etaient exclus de l’étude les patients qui avaient présenté une cataracte bilatérale homogène et ceux ayant une perception lumineuse négative (même s’il y a une notion de traumatisme oculaire).

### Traitement chirurgical

Les techniques classiques de l’extraction extracapsulaire ou intracapsulaire avec ou sans implantation, ont été pratiquées par le même chirurgien, que ce soit en poste fixe au centre de référence régional (Centre Hospitalier Régional de Sokodé) ou en stratégie avancée dans les chefs-lieux des districts sanitaires de la Région Centrale. Les implants standards de type PMMA (AUROLAB) de 20 à 23 dioptries selon la disponibilité, ont été utilisés. Le suivi post opératoire était réalisé dans chaque centre systématiquement au premier jour post opératoire (J1), puis de la première semaine (S1) à S8 et plus par des techniciens supérieurs en ophtalmologie (TSO) ou par le médecin ophtalmologiste. Tous les patients avaient bénéficié d’une anesthésie locale rétrobulbaire classique. Seuls les enfants âgés de moins de 12 ans avaient été opérés sous anesthésie générale. Les résultats fonctionnels obtenus à la suite de la mesure de l’acuité visuelle sans correction et avec correction ont permis de classer les patients en nous inspirant des recommandations de l’Organisation Mondiale de la Santé (OMS) en trois catégories: les bons résultats (acuité visuelle ≥3/10); les résultats moyens (acuité visuelle comprise entre 1/10 et 3/10) et les mauvais résultats (acuité visuelle < 1/10).

### Variables étudiées

Les variables étudiées étaient l’Acuité Visuelle initiale (AV), l’AV post opératoire sans correction (AVSC) et avec correction (AVAC), le type de chirurgie, les complications et le suivi post opératoire. Les données recueillies ont été saisies, contrôlées et analysées dans le logiciel Epi Info version 6.4.

## Résultats

### Aspects généraux

Cent trente un (131) dossiers de patients opérés de cataractes et répondant à nos critères dans les quatre districts périphériques et le centre de référence, ont été dépouillés. Au premier jour post-opératoire, la mesure des acuités visuelles sans correction (AVSC) n’avait pas été reportée sur six dossiers, ramenant notre échantillon à 125 patients. Parmi eux, 10 n’avaient pas bénéficié de correction, ramenant ainsi l’échantillon à 115. Environ 70% des patients ont été opérés en stratégie avancée. Le sex - ratio était de 2,54 en faveur du sexe masculin. La moyenne d’âge était de 30,01 ± 16,61 ans avec des extrêmes de 5 et 70 ans. Le Tableau 1 représente la répartition des patients selon les tranches d’âges et le sexe

**Tableau 1:** Répartition des patients opérés selon les tranches d’âges (ans) et le sexe

	F	M	Total	%
5- 14 ans	7	17	24	18,32
15- 24 ans	12	24	36	27,48
25- 34 ans	7	19	26	19,84
35- 44 ans	3	16	19	14,50
45- 54 ans	4	8	12	9,16
55- 64 ans	0	7	7	5,35
65 ans et +	4	3	7	5,35
**Total**	**37**	**94**	**131**	**100,00**

### Aspects thérapeutiques

**Acuité Visuelle initiale (AV):** sur 131 patients, 95% avaient une acuité visuelle initiale limitée à la perception lumineuse. Parmi, les 5% restants, deux patients pouvaient compter les doigts et pour le reste des patients aucune précision n’avait été faite dans leurs dossiers.

**La technique chirurgicale:** la technique classique de l’extraction extra-capsulaire avec implant de chambre postérieure a été pratiquée chez 80% des patients. Quatre-vingt-cinq pourcent (85%) des patients avaient bénéficié des implants standards (80% en chambre postérieure et 5% en chambre antérieure). Quatorze pourcent (14%) seulement des patients ont bénéficié d’une anesthésie générale contre 86% d’anesthésie rétrobulbaire.

**Complications:** sur les 131 patients opérés, 22 (17%) avaient présenté des complications per opératoires à type d’issue de vitré. Les complications post opératoires au cours du suivi avaient concerné 19 patients (15%) : 11 œdèmes cornéens, 05 inflammations majeures, 02 hernies de l’iris et un hyphéma.

### Résultats fonctionnels

**Figure 1:**
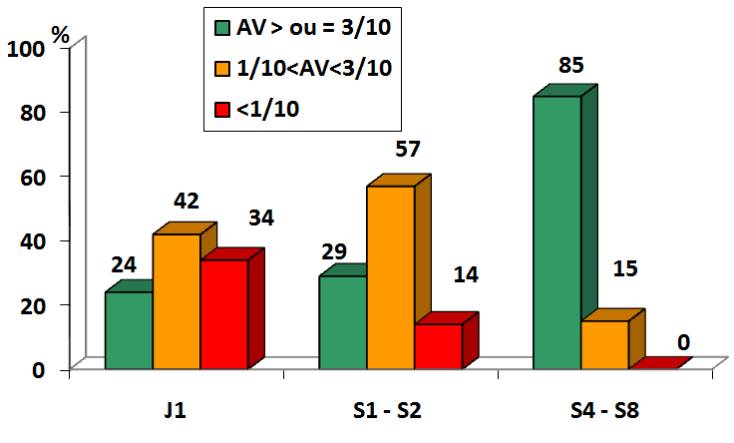
Répartition des patients opérés selon les AV avec correction (AVAC)

La Figure 1 résume la répartition des patients selon les AV post opératoires avec correction(AVAC). Le suivi avait été fait au premier jour post opératoire (J1) puis toutes les semaines jusqu’à la huitième semaine (S1 à S8). Sous correction optique, les résultats étaient bons dans 85% des cas au bout de deux mois. Les 34% de mauvais résultats fonctionnels observés à J1 post opératoire ainsi que les 57% de moyens résultats à S1-S2 ont évolué favorablement s’annulant pour les premiers et se réduisant à 15% pour les seconds. En l’absence de correction, l’AV mesurée des patients est renseignée par le Tableau 2. L’AVSC était supérieure à 3/10^ème^ dans 55% des cas à deux mois. Globalement, il a été constaté une chute régulière du nombre de patients opérés suivis de S1 (41 cas) à S8 (20 cas).

**Tableau 2:** Répartition des patients opérés selon les AV sans correction (AVSC)

	AV SC J1		AV SC S1		AV SC S1- S2		AV SC S2- S4		AV SC S4 - S8	
10/10 - 3/10	14	11%	8	20%	3	8%	7	22%	11	55%
‹ 3/10 - 1/10	55	44%	23	56%	24	67%	19	59%	8	40%
‹ 1/10	56	45%	10	24%	9	25%	6	19%	1	5%
**Total**	**125**	**100%**	**41**	**100%**	**36**	**100%**	**32**	**100%**	**20**	**100%**

## Discussion

### Les limites de l’étude

Cette étude est basée sur l’analyse des dossiers et comptes rendus opératoires des patients ayant bénéficié d’une prise en charge chirurgicale pour cataracte traumatique sur une période de 30 mois allant de janvier 2009 à juin 2011. Elle s’est alors confrontée au problème récurrent de la bonne tenue des dossiers des patients. Ensuite près des trois quarts des patients ont été opérés en stratégie avancée dans des centres distants parfois de 80km du centre de référence; et les patients de provenances diverses étaient souvent venus de contrées éloignées, parfois d’accès difficiles. Tout ceci pourrait expliquer en partie les difficultés du suivi régulier des patients opérés qui ne respectaient pas les rendez-vous de contrôle. La plupart de ces patients ont souvent évoqué le manque de moyens financiers pour revenir dans les services de soins oculaires. Ces écueils constituent sans doute des biais qui n’altèrent en rien l’essentiel des résultats de cette étude.

### Aspects thérapeutiques

Sur les 131 patients de notre série, environ 14% avaient au plus 12 ans et avaient bénéficié d’une anesthésie générale. Doutetien et al. [6], ont rapporté un taux plus élevé d’anesthésie générale (26,7%). Ceci serait en relation avec le fait que leur série qui avait une moyenne d’âge plus faible comporterait plus d’enfants en bas âge. En effet, la moyenne d’âge de leur série était de 26 ans avec des extrêmes de 3 ans et 59 ans alors que les nôtres étaient de 5 ans et 70 ans.

Tous les patients ont bénéficié de la chirurgie en différé. La technique classique de l’extraction extra-capsulaire de la cataracte a été la plus pratiquée (95%). Baklouti et al. [7] ont rapporté un taux plus bas avec 90% de même que Doutetien et al. [6] avec un taux de 54,5%. Chuang et Lai [8] à Taiwan dans un centre de référence tertiaire ont rapporté un taux d’implantation de 100% en chambre postérieure, plus élevé que les 80% d’implantation en chambre postérieure de la présente série. Ces auteurs avaient pratiqué la fixation sclérale de l’implant dans les cas de rupture de la capsule postérieure. Signalons que dans notre cas 10% des cataractes traumatiques étaient sub-luxées et pas faciles à implanter.

### Aspects fonctionnels

Pour analyser les résultats post opératoires, nous nous sommes inspirés de la classification de l’OMS sur les résultats fonctionnels de la chirurgie de la cataracte en général. Il faut préciser que dans cette classification, l’OMS recommande de ne pas tenir compte des cataractes traumatiques et des patients opérés âgés de moins de 20 ans. Cependant, à défaut d’un repère adapté, nous nous sommes néanmoins référés à ce dernier. De nos résultats, il est ressorti qu’environ 66% des patients présentaient à J1 post opératoire une AV≥1/10 quittant ainsi l’état de cécité dans lequel ils se trouvaient auparavant. Au -delà de deux mois de suivi, seulement 20 patients (17%) sont revenus pour le suivi post opératoire. Parmi eux, après correction, 17 (85%) présentaient de bons résultats avec une AV≥3/10 pendant que les mauvais résultats (AV<1/10) étaient réduits à néant. Doutetien et al. [6] ont rapporté un résultat légèrement plus bas car sur 14 yeux suivis, 11 (78,5%) ont atteint une AV corrigée supérieure à 3/10 (bons résultats).

## Conclusion

Malgré les progrès de la prise en charge microchirurgicale des traumatismes oculaires en général et de la cataracte traumatique en particulier, cette dernière demeure difficile à la fois pour le patient et le praticien. Ces difficultés augmentent dans les pays en développement qui disposent de ressources très limitées. Néanmoins, il est possible dans ces contextes d’obtenir des résultats encourageant. L’inexistence d’un standard de suivi des résultats fonctionnels des cataractes traumatiques opérées à l’instar de celui proposé par l’OMS demeure une difficulté. L’élaboration de ce standard combinée à l’accessibilité aux soins des communautés devraient améliorer la prise en charge des cataractes traumatiques.

### Etat des connaissances actuelles sur le sujet

- Fréquence des cataractes traumatiques chez les sujets en pleine activité;
- Compromet gravement la fonction visuelle surtout chez les enfants avec le risque d’amblyopie;
- Mauvais résultats fonctionnels malgré la microchirurgie et d’autant plus dans les pays en développement ou l’accès à la chirurgie oculaire en zone rurale est faible.

### Contribution de notre étude à la connaissance

- Rapprochement de la chirurgie des cataractes traumatiques des localités lointaines par la stratégie avancée;
- Former et associer les paramédicaux (TSO techniciens supérieurs d’Ophtalmologie) dans le suivi post opératoire de la chirurgie de la cataracte pour optimiser les résultats surtout dans la stratégie avancée dans les milieux très éloigné;
- Malgré les moyens limités la prise en charge de ces cataractes a donné des résultats très encourageants et permit de sortir les patients de la cécité.
